# Novel *Chlamydiales* genotypes identified in ticks from Australian wildlife

**DOI:** 10.1186/s13071-017-1994-y

**Published:** 2017-01-26

**Authors:** Delaney Burnard, Haylee Weaver, Amber Gillett, Joanne Loader, Cheyne Flanagan, Adam Polkinghorne

**Affiliations:** 1 Centre for Animal Health Innovation, Faculty of Science, Health, Education and Engineering, University of the Sunshine Coast, Sippy Downs, QLD 4556 Australia; 2 Australian Government, Department of Environment and Energy, Australian Biological Resources Study, GPO Box 787, Canberra, ACT 2601 Australia; 3 Australia Zoo Wildlife Hospital, Steve Irwin Way, Beerwah, QLD 4519 Australia; 4 Endeavour Veterinary Ecology Pty Ltd, 1695 Pumicestone Rd, Toorbul, QLD 4510 Australia; 5 Port Macquarie Koala Hospital, Roto House Historic Site, Cnr Lord Street and Roto Place, Port Macquarie, 2444 NSW Australia

**Keywords:** *Chlamydia*, *Chlamydia*-like organisms, Ticks, Marsupials, Wildlife, Transmission, Vector, Australia

## Abstract

**Background:**

Members of the order *Chlamydiales* are known for their potential as human and veterinary bacterial pathogens. Despite this recognition, epidemiological factors such as routes of transmission are yet to be fully defined. Ticks are well known vectors for many other infections with several reports recently describing the presence of bacteria in the order *Chlamydiales* in these arthropods. Australian wildlife are hosts to an extensive range of tick species. Evidence is also growing that the marsupial hosts these ticks parasitise can also be infected by a number of bacteria in the order *Chlamydiales*, with at least one species, *Chlamydia pecorum*, posing a significant conservation threat. In the current study, we investigated the presence and identity of *Chlamydiales* in 438 ixodid ticks parasitizing wildlife in Australia by screening with a pan-*Chlamydiales* specific targeting the 16S rRNA gene.

**Results:**

Pan-*Chlamydiales* specific PCR assays confirmed the common presence of *Chlamydiales* in Australian ticks parasitising a range of native wildlife. Interestingly, we did not detect any *Chlamydiaceae*, including *C. pecorum*, the ubiquitous pathogen of the koala. Instead, the *Chlamydiales* diversity that could be resolved indicated that Australian ticks carry at least six novel *Chlamydiales* genotypes. Phylogenetic analysis of the 16S rRNA sequences (663 bp) of these novel *Chlamydiales* suggests that three of these genotypes are associated with the *Simkaniaceae* and putatively belong to three distinct novel strains of *Fritschea* spp. and three genotypes are related to the “*Ca*. Rhabdochlamydiaceae” and putatively belong to a novel genus, Rhabdochlamydia species and strain, respectively.

**Conclusions:**

Sequence results suggest Australian wildlife ticks harbour a range of unique *Chlamydiales* bacteria that belong to families previously identified in a range of arthropod species. The results of this work also suggest that it is unlikely that arthropods act as vectors of pathogenic members of the family *Chlamydiaceae*, including *C. pecorum*, in Australian wildlife. The biology of novel *Chlamydiales* identified in arthropods remain unknown. The pathogenic role of the novel *Chlamydiales* identified in this study and the role that ticks may play in their transmission needs to be explored further.

**Electronic supplementary material:**

The online version of this article (doi:10.1186/s13071-017-1994-y) contains supplementary material, which is available to authorized users.

## Background

Bacteria of the order *Chlamydiales* are obligate intracellular parasites. Many of the best described members of this order belong to the traditional family *Chlamydiaceae*, well known for causing significant disease in humans and a variety of domesticated and wild animals [1]. Outside of this family, four other families are taxonomically recognised in this order, the *Waddliaceae*, *Parachlamydiaceae*, *Criblamydiaceae* and *Simkaniaceae* [2–4]. There is also a growing recognition of the broader biodiversity within this order with the relatively recent proposal of four additional families; “*Candidatus* Parilichlamydiaceae” [5], “*Ca*. Piscichlamydiaceae” [6], “*Ca*. Clavichlamydiaceae” [7] and “*Ca*. Rhabdochlamydiaceae” [8] that are yet to be accepted. These recently proposed families are largely comprised of bacteria that, to date, predominantly infect fish (Parilichlamydiaceae, Piscichlamydiaceae, Clavichlamydiaceae) or invertebrates (Rhabdochlamydiaceae).

Contrary to the obligate intracellular nature of the chlamydial developmental cycle, many species in the order *Chlamydiales* can infect multiple hosts across a diverse range of taxa [9]. Beyond the well-described zoonotic potential of the avian pathogen, *Chlamydia psittaci* [10], perhaps one of the best known species with these capabilities is *Chlamydia pecorum*, a highly prevalent veterinary pathogen. Globally, *C. pecorum* infects livestock such as sheep, cattle, pigs and goats, and the iconic and nationally vulnerable marsupial, the koala, in Australia [11, 12]. In these hosts, these infections are primarily asymptomatic but can lead to diseases such as pneumonia, arthritis, keratoconjunctivitis and encephalomyelitis in livestock [12] and keratoconjuctivitis, cystitis and reproductive disease in koalas [11]. While within-host routes of transmission are reasonably well characterised for members of the family *Chlamydiaceae*, much less is known about the transmission of bacteria in other chlamydial families or how cross-host transmission events occur for these pathogens. For the latter, direct contact with infected animals is a risk factor for zoonotic transmission of *C. psittaci* [13] and *Chlamydia abortus* [14] while indirect contact *via* environmental contamination from infected animals is also suspected for the former species [15]. Other potential routes of transmission including the use of vectors are largely unknown.

Ticks (Acari: Ixodida) are known to be important vectors in transmitting microorganisms capable of infecting both human and animal hosts. Their ability to feed on such a large range of host species [16] places ticks second to mosquitoes in pathogen transmission worldwide [17]. Studies in the 1970–1980’s suggested that *Chlamydia* may be a naturally occurring pathogen carried by ticks, through experimental infection studies that described multiplication followed by transmission and disease manifestation in cattle [18, 19]. Further studies were not performed until recently when *Chlamydiaceae* were identified as dominant taxa in the microbiomes of Japanese ticks, with several *Parachlamydia* and *Simkaniaceae* sequences identified [20]. Subsequently, seven *Chlamydiales* families have been identified across large scale tick screens in Switzerland, Algeria and Finland [21–23]. The most common *Chlamydiales* detected in these studies were “*Ca.* Rhabdochlamydia”, “*Ca*. Parachlamydia” and other unclassified and uncultured *Chlamydiales*, suggesting that the diversity of *Chlamydia* and *Chlamydia*-like organisms (CLOs) carried and potentially transmitted by tick species is large and unique.

Seventy tick species have been described in Australia, including 14 soft and 56 hard ticks. Five of these species are suspected of being introduced by humans. Fifty four of these 70 tick species solely feed on wildlife, whereas 16 tick species are known to feed on humans and domestic animals also [24]. Very little is known about Australian ticks in regards to the pathogens they harbour and their potential to transmit these pathogens to humans and wildlife. As of now, only five tick species are known to carry and transmit pathogens including, Queensland tick typhus (*Rickettsia australis*), transmitted by *Ixodes holocyclus* and *Ixodes tasmani* [25]; Flinders Island spotted fever (*Rickettsia honei*) transmitted by *Bothriocroton hydrosauri* [26]; as well as Q fever (*Coxiella burnetii*), previously detected in tick species *Haemaphysalis humerosa* [27] and *Amblyomma t. triguttatum* [28]. Novel bacteria belonging to the *Rickettsia*, *Coxiella*, *Rickettsiella* and *Borrelia* have recently been described from native Australian ticks removed from Australian marsupials [29, 30], suggesting the majority of bacterial diversity of Australian ticks is yet to be discovered.

As mentioned previously, *Chlamydia* is prevalent in Australian wildlife [11, 24] and there is also evidence to suggest CLOs have a presence in native marsupials [31–33]. To date, nothing is known about the prevalence of *Chlamydia* and CLOs in Australian ticks or if they have the potential to vector transmit *Chlamydiales* between wildlife and other hosts. In the present study, we identified and screened Australian tick species removed from native Australian wildlife hosts to assess the role that these arthropods may have in the transmission of chlamydial infections between Australian wildlife.

## Methods

### Tick collection, identification and pooling

Ticks were opportunistically removed from marsupials and monotremes presenting to collaborating veterinarian and wildlife care centres in New South Wales, Queensland and Tasmania, Australia and stored in 70% ethanol. Macroscopic residual host tissue collected with the tick during sampling was removed using forceps and 70% ethanol washing. Morphological characteristics were then used to identify tick species [34, 35]. Prior to DNA extraction, ticks were then sorted by species and animal host and pooled (1–5 ticks per pool).

### Tick dissection and DNA extraction

Tick dissection and DNA extraction was modified from a previously described QIAGEN quadrisecting protocol [36], to account for the size and density of heavily engorged ticks. Individual ticks in a pool were rinsed twice with dH_2_O, then air dried for 10 min. Dissections were carried out by cutting anteriorly to posteriorly down the middle of the hypostome then on a 90 degree angle directly under coxa IV. This tick section was then finely diced and re-pooled into a microcentrifuge tube with sections from the remaining ticks in each pool for DNA extraction. Sterile instruments were used for each individual dissection. Using the QIAmp DNA mini kit (QIAGEN, Victoria, Australia) tick pools were lysed in 260 μl ATL buffer and 20 μl Proteinase K at 56 °C for 48 h vortexing occasionally and the DNA extraction was completed using the ‘DNA purification from tissues’ protocol as per the manufacturer’s instructions. DNA was stored at -20 °C until further use.

### *Chlamydiales* PCR amplification, purification and sequencing

An 800 bp fragment of the 16S rRNA gene partially covering the *Chlamydiales* signature sequence was amplified by conventional PCR to screen ticks for *Chlamydiales* [5, 6]. The reaction was made up to a final volume of 50 μl containing 25 μl of Amplitaq Gold 360 master mix (Life Technologies, Victoria, Australia), 1.5 μl of each primer 16SIGF ( 5′-CGG CGT GGA TGA GGC AT-3′) and 806R (5′-GGA CTA CCA GGG TAT CTA AT-3′) and 4 μl of template DNA. Thermocycler conditions used were as previously described [5]. Negative (no template and dH_2_O) and positive (*C. pecorum* PM13 cultured isolate from an Australian koala) controls were included in each amplification assay. At least 50 16S rRNA copies of chlamydial DNA can be reliably detected with this assay (data not shown). PCR product was purified using the Roche High Pure PCR Product Purification Kit (Roche, New South Wales, Australia) following the manufacturer’s instructions. Purified PCR products were di-deoxy sequenced by Macrogen Inc. (Seoul, Korea) in both directions.

### Sequence and phylogenetic analysis

Chromatograms of forward and reverse sequences were aligned in the Geneious R9.1.3 software package [37]. A consensus sequence was derived from each alignment and trimmed to the maximum length possible. Consensus sequences and GenBank representatives of the order *Chlamydiales* were then aligned in Geneious R9.1.3 using the ClustalW plugin with default parameters [38]. The alignment comprised 44 sequences trimmed to a length of 663 bp and a Bayesian phylogeny was constructed in Geneious R9.1.3 using the MrBayes plugin [39] under the HKY85 substitution model. Run parameters included four Markov chain Monte Carlo (MCMC) chains with a million generations, sampled every 3,000 generations, and with the first 100,000 trees discarded as ‘burn-in’.

### Predicted prevalence of *Chlamydiales* infections of individual ticks

*Chlamydiales* prevalence for individual ticks was calculated using maximum likelihood estimates with a confidence interval of 0.95, following previously described methods [21]. Pool size and pool PCR positivity data are used to estimate an individual *Chlamydiales* prevalence in ticks as well as a minimum and maximum prevalence rate.

## Results

### Tick species identified from Australian wildlife

Overall, 438 adult female engorged ixodid ticks comprised of seven species were identified from ten marsupial hosts and one monotreme host species. Adult female ticks were the most prevalent tick opportunistically removed from animals in this study. The number of male ticks and nymphs removed from animals was unfortunately limited. Due to the low numbers and difficulties in morphological identification to species of male ticks and nymphs, these specimens were excluded from this study. The wildlife host and tick species, number and location (Australian state) are presented in Additional file 1: Table S1. The most common tick species identified from koalas was *I. tasmani* (300/438; 68.5%) and *I. holocyclus* (100/438; 21.9%). Greater tick species diversity was identified from non-koala marsupial hosts, with *I. holocyclus* being the most common, followed by *Haemaphysalis bancrofti*.

### Prevalence of *Chlamydiales* in Australian ticks

The 438 adult female engorged Ixodidae ticks were distributed into a total of 124 pools ranging between one to five ticks based on tick species and wildlife host, then screened using a pan-*Chlamydiales* PCR (Table 1). Of these 124 pools, 99 were found to be positive for *Chlamydiales* DNA. Based on this result, individual *I. tasmani* ticks have an estimated *Chlamydiales* prevalence of 26.9% (minimum prevalence: 17.4%, maximum prevalence: 71.6%), while individual *I. holocyclus* ticks were estimated to have a *Chlamydiales* prevalence of 46.8% (minimum prevalence: 33%, maximum prevalence: 61.9%). Of these species collected from koalas, the estimated prevalence of individual ticks was 24.0% (minimum prevalence: 15.3%, maximum prevalence: 73.5%). All other tick species and wildlife hosts were 100% PCR positive for *Chlamydiales* DNA, although the numbers screened were significantly smaller than for the other two tick species.

**Table 1:** Estimated prevalence of *Chlamydiales* infections in ixodid ticks removed from Australian wildlife based on a maximum likelihood analysis

	No. of ticks	No. of pools	No. of PCR positive pools (%)	Estimated prevalence in individual ticks %	Minimum prevalence (%)^a^	Maximum prevalence (%)^b^
Tick species						
*I. tasmani*	310	73	54 (73.9)	26.9	17.4	71.6
*I. holocyclus*	112	35	29 (82.9)	46.8	33	61.9
*I. ornithorhynchi*	2	2	2 (100)	na	na	na
*H. bancrofti*	10	10	10 (100)	na	na	na
*H. longicornis*	1	1	1 (100)	na	na	na
*H. humerosa*	1	1	1 (100)	na	na	na
*B. auruginans*	2	2	2 (100)	na	na	na
Wildlife host						
Koala (*Phascolarctos cinereus*)	400	86	61 (70.9)	24	15.25	73.5
Bare nosed wombat (*Vombatus ursinus*)	5	5	5 (100)	na	na	na
Eastern grey kangaroo (*Macropus giganteus*)	9	9	9 (100)	na	na	na
Red necked wallaby (*Macropus rufogriseus*)	3	3	3 (100)	na	na	na
Spotted tail quoll (*Dasyurus maculatus*)	4	4	4 (100)	na	na	na
Long nosed bandicoot (*Perameles nasuta*)	3	3	3 (100)	na	na	na
Platypus (*Ornithorhynchus anatinus*)	2	2	2 (100)	na	na	na
Squirrel glider (*Petaurus norfolcensis*)	2	2	2 (100)	na	na	na
Short eared possum (*Trichosurus caninus*)	1	1	1 (100)	na	na	na
Brush tail possum (*Trichosurus vulpecula*)	7	7	7 (100)	na	na	na
Ring tailed possum (*Pseudocheirus peregrinus*)	2	2	2 (100)	na	na	na

*Abbreviation*: *na* not applicable; pools only consisted of one individual tick

^a^ The minimum prevalence in individual ticks is calculated on the assumption only one individual tick per *Chlamydiales* positive pool is infected

^b^ The maximum individual prevalence is similarly calculated on the assumption every individual tick in a PCR positive pool is infected

### *Chlamydiales* detected in Australian ticks

To determine the identity of the *Chlamydiales* detected in the ticks screened in this study, the PCR products of 66 pan-*Chlamydiales* PCR positive pools were directly sequenced. 53/66 (80.3%) of the PCR products returned clear sequencing signals to enable BLAST analysis against all available *Chlamydiales* 16S rRNA gene sequences available in GenBank. Chromatograms for the remaining PCR positive samples were unreadable, despite repeated PCR amplification and sequencing efforts, suggesting multiple *Chlamydiales* agents are present in these tick pools.

The resulting BLAST analysis revealed 53 chlamydial 16S rRNA gene sequences, comprised of six novel 16S rRNA *Chlamydiales* genotypes consistent with new strains, species or genera within the order *Chlamydiales*, based on previously described taxonomic cut-offs specific to the order *Chlamydiales* [2, 40] (Table 2). Interestingly, we did not identify any 16S rRNA sequences that were identical to those previously described for species in the order *Chlamydiales*, most notably for members of the *Chlamydiaceae,* such as *C. pecorum*, a species known to infect the hosts that these ticks were removed from.

**Table 2:** Abundance of novel *Chlamydiales* genotypes in tick species and tick hosts

Genotype	Tick species (No.)	Tick host (No.)	BLAST ID (% similarity)
1	*I. tasmani* (23); *I. holocyclus* (2); *I. ornithorhynchi* (1)	Koala (24); Brushtail possum (1); Platypus (1)	“*Ca*. Rhabdochlamydiaceae porcellionis” AY223862.1 (96%)
2	*I. tasmani* (7); *I. holocyclus* (3); *I. ornithorhynchi* (1); *H. bancrofti* (5); *H. longicornis* (1)	Koala (6); Red necked wallaby (1); Brushtail possum (2); Platypus (1); Eastern grey kangaroo (5); Bare nosed wombat (1); Ringtailed possum (1)	“*Ca*. Fritschea eriococci” AY140911.1 (99%)
3	*I. tasmani* (4); *I. holocyclus* (1)	Koala (5)	“*Ca*. Rhabdochlamydiaceae porcellionis” AY223862.1 (98%)
4	*I. tasmani* (1); *H. humerosa* (1)	Long nose bandicoot (2)	“*Ca*. Fritschea eriococci” AY140911.1 (99%)
5	*I. holocyclus* (2)	Spotted tail quoll (1); Brushtail possum (1)	“*Ca*. Fritschea eriococci” AY140911.1 (99%)
6	*I. tasmani* (1)	Koala (1)	“*Ca*. Rhabdochlamydiaceae” bacterium FJ976099.1 (92%)

**Fig. 1:**
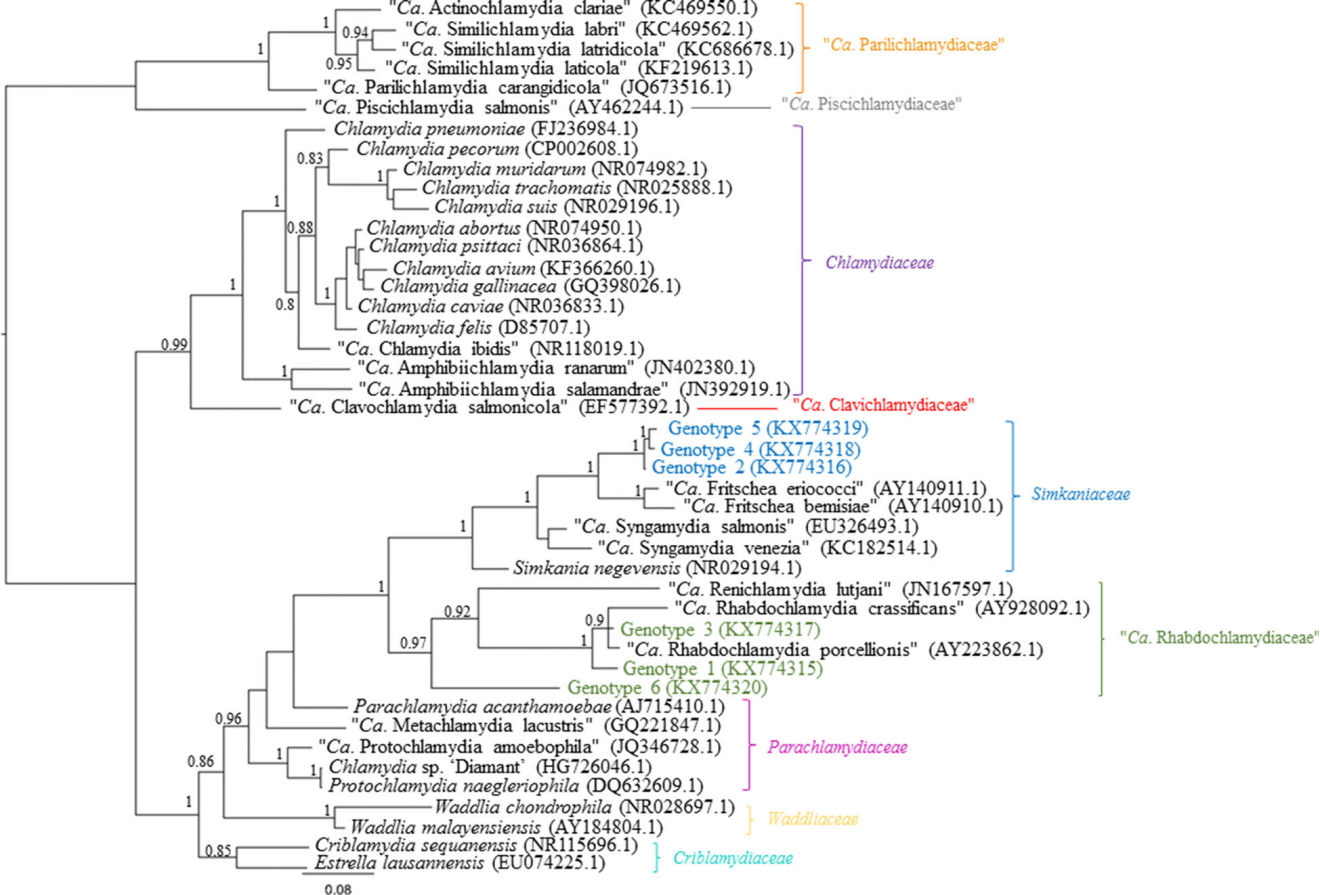
Phylogenetic relationships of the novel *Chlamydiales* genotypes identified in Australian ticks. Bayesian tree incorporating representative 16S rRNA sequences of each family of the order *Chlamydiales* from GenBank, as well as the six novel 16S rRNA genotypes identified in this study. Tree was built using a 663 bp under the HKY85 evolutionary model, posterior probability exceeding 0.75 is shown at internal nodes

Based on BLAST and subsequent phylogenetic analysis (Fig. 1), the novel 16S rRNA *Chlamydiales* genotypes identified in this study were predicted to be associated with the “*Ca*. Rhabdochlamydiaceae” and *Simkaniaceae* families. The most prevalent of these sequences, Genotype 1 (KX774315), represented nearly half (26/53; 49.1%) of all the sequences retrieved and was identified in ticks of the genus *Ixodes* (*I. tasmani*, *I. holocyclus* and *I. ornithorhynchi*) from koala, brushtail possum and platypus hosts. BLAST comparisons revealed that this genotype has 96% similarity to “*Ca*. Rhabdochlamydia porcellionis” (AY223862.1), suggesting that, based on current taxonomic classifications, it belongs to a previously undescribed species within the genus “*Ca*. Rhabdochlamydia”. Consistent with this BLAST analysis, this sequence clustered within the “*Ca*. Rhabdochlamydiaceae”, but formed an independent novel lineage from “*Ca*. Rhabdochlamydia porcellionis” with posterior probability (pp) support of 1.

The second most prevalent of these sequences was Genotype 2 (KX774316), which represented just under a third (17/53; 32%) of all the sequences retrieved. Genotype 2 was identified in ticks of the genera *Ixodes* and *Haemaphysalis* (*I. tasmani*, *I. holocyclus*, *I. ornithorhynchi*, *H. longicornis* and *H. bancrofti*) from koalas and a variety of other marsupials (brushtail possum, red necked wallaby, eastern grey kangaroo, ringtail possum and wombat) and monotremes (platypus) examined. BLAST comparisons revealed this genotype has 99% similarity to “*Ca*. Fritschea eriococci” (AY140911.1), indicating that the sequences detected potentially belong to strains related to this previously described chlamydial species. This relationship is demonstrated in the phylogeny where there is strong support (pp 1) for Genotype 2 branching off “*Ca*. Fritschea eriococci” (AY140911.1) as a distinct lineage (Fig. 1).

The third most prevalent of these sequences was Genotype 3 (KX774317), representing almost one fifth (5/53; 9.4%) of the sequences retrieved. This particular genotype was identified only from *I. tasmani* and *I. holocyclus* removed from koalas. BLAST comparisons revealed this genotype has 98% similarity to “*Ca*. Rhabdochlamydia porcellionis” (AY223862.1), which would suggest that these sequences belong to a previously undescribed strain belonging to “*Ca*. Rhabdochlamydia porcellionis”. Phylogenetically, this novel genotype sits between two previously proposed species in the genus “*Ca*. Rhabdochlamydia” (pp 0.9; Fig. 1).

Like Genotype 2, Genotypes 4 (KX774318) and 5 (KX774319) were also found to have 99% similarity to “*Ca*. Fritschea eriococci” (AY140911.1), following BLAST analysis, indicating that the sequences detected potentially belong to strains related to this previously described chlamydial species. Less than 1% dissimilarity separate these three novel 16S rRNA genotypes with four single nucleotide polymorphisms found between Genotypes 2 and 4 and five single nucleotide polymorphisms between Genotype 5 and Genotypes 2 and 4. The relationship between Genotypes 2, 4 and 5 to “*Ca*. Fritschea eriococci” (AY140911.1) was strongly supported (pp 1) in the phylogeny (Fig. 1). Genotype 4 was identified in only two (3.7%) of all the sequences retrieved, one from an *I. tasmani* and one from an *H. humerosa* tick, both removed from long nosed bandicoot hosts exclusively. Genotype 5 was identified in two sequences (2/53; 3.7%) overall, from two *I. holocyclus* ticks removed from a spotted tail quoll and brushtail possum, respectively.

Genotype 6 (KX774320), was identified from only one pool of *I. tasmani* ticks removed from koalas. BLAST comparisons revealed this genotype has 92% similarity to “*Ca*. Rhabdochlamydia porcellionis” (AY223862.1), consistent with it coming from a novel bacteria in a new genus that is closely related to the “*Ca*. Rhabdochlamydia”. This genus-level relationship was strongly supported in the phylogeny (pp 0.97; Fig. 1).

### Genotype diversity across tick species

**Fig. 2:**
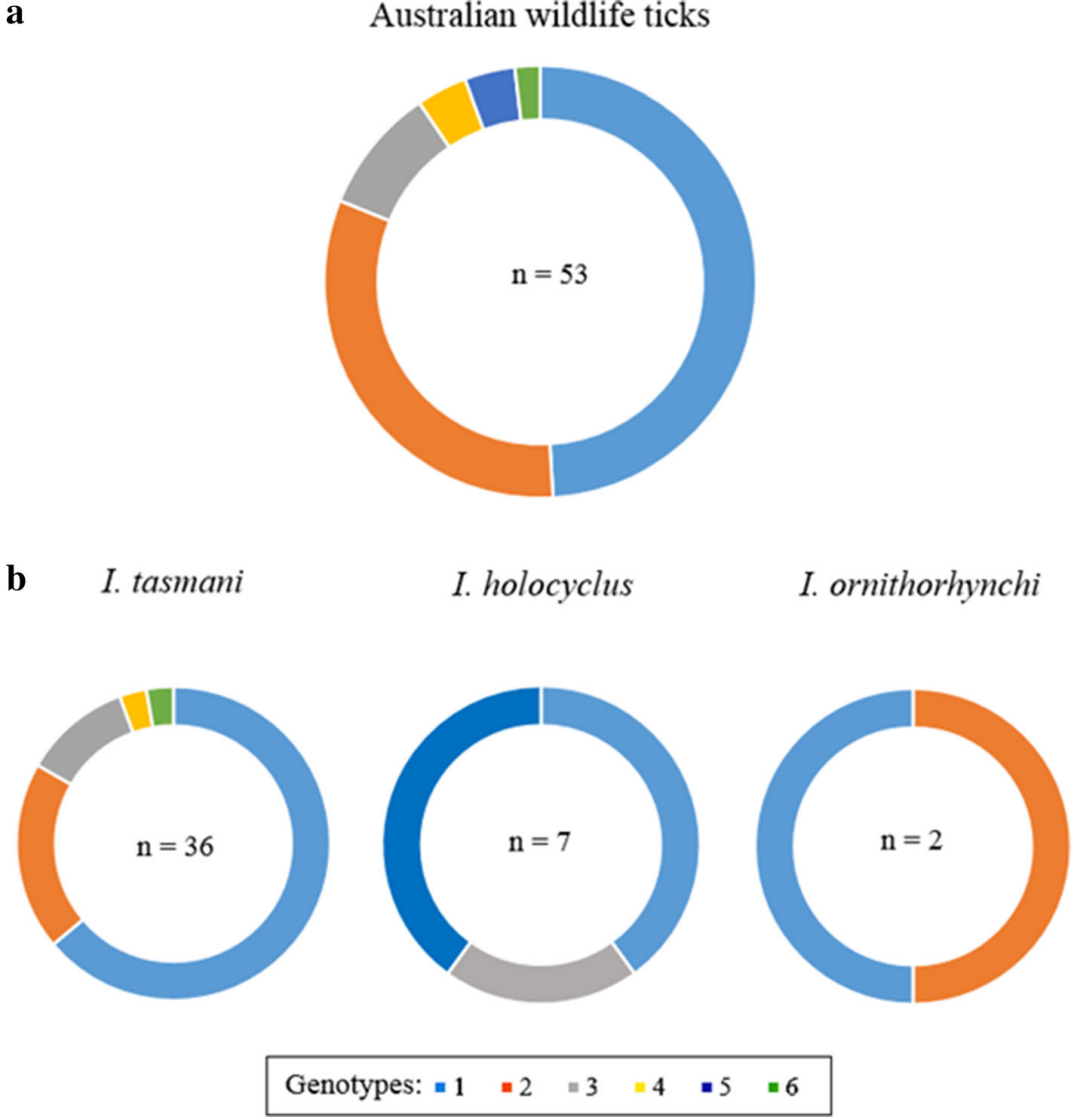
Distribution of novel *Chlamydiales* genotypes amongst Australian ticks. **a** Doughnut chart displays the number of sequences retrieved from each *Chlamydiales* genotype within Australian ticks removed from native wildlife. **b** Doughnut charts displays the number of sequences retrieved from each *Chlamydiales* genotype between Australian tick species removed from wildlife that cumulatively carried more than one genotype

An analysis of the tick host range of these novel *Chlamydiales* revealed that tick species within the genus *Ixodes* harbour a diverse range of novel *Chlamydiales* (Fig. 2), whereas ticks of the genus *Haemaphysalis* (*H. bancrofti*, *H. longicornis* and *H. humerosa*) each only carried one novel chlamydial genotype (Fig. 2). The most prevalent koala tick species *I. tasmani,* carried the most genotypic diversity with five of the six genotypes cumulatively identified in this species, with Genotype 1 dominating the sampling, totalling 63% of the diversity. *Ixodes holocyclus*, the second most prevalent koala tick species and the most prevalent non-koala marsupial tick species carried three of the six novel genotypes, all with a relatively equal abundance. The two platypus ticks, *I. ornithorhynchi* both carried a different genotype.

## Discussion

Australian ticks have previously been found to be a rich reservoir of novel bacteria belonging to otherwise well-known families of bacterial pathogens. A pan-*Chlamydiales* order specific 16S rRNA PCR screening strategy provided molecular evidence that Australian ticks parasitising native Australian wildlife carry a diverse range of novel bacteria from the order *Chlamydiales*.

The results of this study indicate that, like previous studies of ticks from Switzerland, Algeria and Finland [21–23], Australian tick species are common hosts for the order *Chlamydiales.* The percentage of *Chlamydiales* PCR positive pools and estimated individual prevalence rates in adult tick species *I. tasmani* and *I. holocyclus* were significantly higher than that described in previous large-scale investigations of ticks performed in Switzerland [21, 22] and Finland [23], however. Although not representative given the limited sample sizes, the *Chlamydiales* prevalence in the Australian ticks examined here was also significantly higher in the five individually screened tick species with 100% positivity, compared with individually screened Algerian ticks with only 45% positivity [22]. These findings suggest that *Chlamydiales* infections are present in Australian tick species with the likelihood that more than one tick per pool is infected with *Chlamydiales*. As the majority of the sequences retrieved were of high quality, it is reasonable to suggest that when a pool is positive, more often than not, multiple individual ticks within that pool are infected with the same *Chlamydiales* bacteria. In order to estimate the true prevalence and rate of infection, a larger sample size and individual tick screening is required.

Interestingly, only novel *Chlamydiales* 16S rRNA genotypes were retrieved from the seven Australian tick species screened in this study. Of the six novel 16S rRNA genotypes identified, three were associated with the family “*Ca*. Rhabdochlamydiaceae” and three with the family *Simkaniaceae*. Whereas in previous tick studies, molecular evidence for six *Chlamydiales* families was retrieved from two Switzerland surveys, five *Chlamydiales* families from the Finnish survey and four *Chlamydiales* families from the Algerian survey [21, 22]. On all three of these occasions, the *Parachlamydiaceae*, “*Ca*. Rhabdochlamydiaceae”, *Criblamydiaceae* families and unclassified *Chlamydiales* were described, with *Parachlamydiaceae* and “*Ca*. Rhabdochlamydiaceae” being the most abundant taxa. Members of the *Simkaniaceae* were also identified in both Switzerland surveys but, in contrast to our study, at a much lower abundance. The differences in *Chlamydiales* composition and prevalence observed in Australian ticks is potentially influenced by four factors: (i) the geographical isolation of Australia, giving rise to a unique evolution of arthropods; (ii) therefore the opportunity for a distinct lineage of Australian *Chlamydiales*; (iii) unique native Australian marsupial and monotreme wildlife, which could harbour their own *Chlamydiales* species; and (iv) the amplification and sequencing approach used is this study. In terms of the latter, a higher resolution of *Chlamydiales* species identity present in ticks was achieved by PCR amplification and sequencing of a longer fragment of the 16S rRNA *Chlamydiales* signature sequence. Previous studies relied upon a smaller 207–215 bp fragment, which only enabled confident identification to the family or genus level, and produced high numbers of unclassified *Chlamydiales*; almost equal to the most prevalent taxa [21, 22]. It is plausible to consider that if additional 16S rRNA gene sequence information had been obtained from these latter screens greater resolution of sequence identity would have been obtained, resulting in higher levels of novel chlamydial diversity, as evidenced in this study.

The identification of multiple diverse “*Ca*. Rhabdochlamydiaceae”-associated genotypes in our ticks is consistent with previous reports for members of this proposed family. The founding members of this family are two species of the genus “*Ca*. Rhabdochlamydia”, “*Ca*. R. porcellionis” and “*Ca*. R. crassificans”, and these two species were identified from arthropods, a woodlouse and cockroach, respectively [8, 41]. The only other member of the family “*Ca*. Renichlamydia lutjani”, was identified in blue stripped snapper [42]. In the most recent Swiss tick screen, “*Ca*. Rhabdochlamydiaceae” were the dominant bacteria in the order *Chlamydiales* identified with chlamydial loads that probably reflect stable replication of these bacteria in their arthropod hosts [21]. The successful amplification and identification of additional novel “*Ca*. Rhabdochlamydiaceae” again in this study suggests that members of this family are primarily bacterial parasites of invertebrate hosts. Highlighting that we have only just begun to reveal the level of biological diversity within this family, as revealed in a recent metagenomic study of the *Chlamydiales* that predicted the “*Ca*. Rhabdochlamydiaceae” to be the most diverse and species rich of the nine known and proposed chlamydial families [43]. While the clinical significance of these bacteria on higher eukaryotes is unclear, further study of the biological diversity within this order, in particular, comparisons of the “*Ca*. Rhabdochlamydiaceae” infecting different *Ixodes* tick species from the northern and southern hemisphere, will provide a rich insight into the evolution and adaptation of CLOs to their arthropod hosts.

In contrast to the presence of the Rhabdochlamydiaceae in ticks in this study, the identification of multiple *Simkaniaceae-*associated genotypes is unexpected given the larger host range of this family. The founding species in this family is the human associated *Simkania negevensis* [44], whereas the two “*Ca*. Syngnamydia” species were identified in fish [45, 46]. Two other proposed species have also been described, “*Ca*. Fritschea eriococci” and “*Ca*. Fritschea bemisiae”, identified from a scale insect and a whitefly insect, respectively [47]. Despite the fact that the *Simkaniaceae* was not a highly abundant family in previous studies [21, 22], our *Simkaniaceae* genotypes made up nearly 40% of sequences resolved in this study with Genotype 2 being the second most prevalent. It was also interesting to note that these genotypes clustered primarily with the previously described arthropod *Simkaniaceae,* suggesting that “*Ca*. Fritschea”-related bacteria, as a subset of the broader diversity in this family, might be primarily arthropod parasites. Downstream detailed biological comparisons between these bacteria and those in the “*Ca*. Rhabdochlamydia” might reveal key mechanisms that are required for survival in arthropods.

Surprisingly, no genus *Chlamydia* sequences were identified from this screen of Australian wildlife ticks despite 400 of these ticks being removed from koalas in areas where *C. pecorum* is considered to be endemic. Based on current data, the *Chlamydiaceae* were only identified in very low numbers in Algerian ticks [22] and as a minor contributor to the dominant taxa in the microbiome of two pools of Japanese ticks, further suggesting the genus *Chlamydia* is not common among arthropods. Observations from a recent Swiss study [21] describe very low loads of *Chlamydiaceae* bacteria (< 10^2^ 16S rRNA copies/μl) compared to loads of up to 10^6^ copies/μl for “*Ca*. Rhabdochlamydiaceae” sequences *via* qPCR, raising questions over whether ticks may be stable hosts for replication of *Chlamydiaceae*. The assay used in this *Chlamydiales* screen can reliably detect chlamydial DNA down to at least 50 16S rRNA copies (data not shown) so it is unlikely that we failed to detect low levels of *Chlamydiaceae* DNA if they were present. Based on our failure to detect *Chlamydiaceae* DNA in any of the tick pools screened in this study, it is unlikely that Australian tick species are acting as vectors of *C. pecorum* between koalas or other animals such as livestock. However, it is interesting to note that there is molecular evidence that native Australian marsupials carry a range of other bacteria in the order *Chlamydiales* [31, 33, 48]. Sequences of the *Parachlamydiaceae* and *Waddliaceae* were identified from non-koala marsupials [31] as well as distinct novel lineage of CLOs from koalas [48]. Furthermore, the Finnish study revealed CLOs from biopsies of human skin with a suspected tick bite were very similar to CLOs carried by Finnish ticks, suggesting that ticks could act as a transmitter of CLOs [23]. Therefore, further investigation into the possibilities of tick to marsupial and marsupial to tick CLO transmission are warranted.

## Conclusions

Novel *Chlamydiales* genotypes were detected in a screen of 438 native Australian ticks. Six novel genotypes were identified and are predicted to represent new levels of taxonomic diversity in the “*Ca*. Rhabdochlamydiaceae”, likely to be the major taxa found in invertebrates and in the *Simkaniaceae,* a previously characterised chlamydial family with bacteria that infect a range of eukaryotic hosts. From this study, it is clear that a unique community of CLOs are present in Australian ticks, however the biology of the novel *Chlamydiales* genotypes identified in this study remains unknown. Comparative studies of these organisms with tick *Chlamydiales* from other geographic regions may provide insight into local adaption and early evolution of these pathogens. Furthermore, no *Chlamydiaceae* were identified from Australian wildlife ticks, suggesting that ticks do not act as a vector transmitter of *Chlamydia* to wildlife and livestock. The role of arthropods as vectors of CLOs needs to be explored further.

## Additional file

Additional file 1: Table S1. Summary of wildlife host and species, number and location of adult female Ixodidae ticks screened for *Chlamydiales* in this study. (DOCX 14 kb)

## Abbreviations

*Ca*.: *Candidatus*
CLOs: *Chlamydia*-like organisms
