# Supplementary material for: Novel Chlamydiales genotypes identified in ticks from Australian wildlife
Source: Parasit Vectors. 2017 Jan 26;10:46. doi: 10.1186/s13071-017-1994-y (PMC5267465; doi:10.1186/s13071-017-1994-y)
Supplement: Additional file 1: Table S1. — Summary of wildlife host and species, number and location of adult female Ixodidae ticks screened for Chlamydiales in this study. (DOCX 14 kb) [file 13071_2017_1994_MOESM1_ESM.docx]

| Wildlife tick host | Ixodid species, (number), state collected |
| --- | --- |
| Koala (*Phascolarctos cinereus*) | *Ixodes tasmani* (300) QLD, NSW  *Ixodes holocyclus* (100) QLD, NSW |
| Bare nosed wombat (*Vombatus ursinus*) | *Bothriocroton auruginans* (2) TAS  *Ixodes tasmani* (3) |
| Eastern grey kangaroo (*Macropus giganteus*) | *Haemaphysalis bancrofti* (9) QLD |
| Red necked wallaby (*Macropus rufogriseus*) | *Ixodes holocyclus* (1) QLD  *Haemaphysalis bancrofti* (1) QLD  *Haemaphysalis longicornis* (1) QLD |
| Spotted tail quoll (*Dasyurus maculatus*) | *Ixodes holocyclus* (4) NSW |
| Long nosed bandicoot (*Perameles nasuta*) | *Ixodes tasmani* (1) QLD  *Ixodes holocyclus* (1) QLD  *Haemaphysalis humerosa* (1) QLD |
| Platypus (*Ornithorhynchus anatinus*) | *Ixodes ornithorhynchi* (2) QLD |
| Squirrel glider (*Petaurus norfolcensis*) | *Ixodes tasmani* (2) QLD |
| Short eared possum (*Trichosurus caninus*) | *Ixodes tasmani* (1) QLD |
| Brush tail possum (*Trichosurus vulpecula*) | *Ixodes tasmani* (1) QLD  *Ixodes holocyclus* (6) QLD |
| Ring tailed possum (*Pseudocheirus peregrinus*) | *Ixodes tasmani* (2) QLD |

**Additional file 1. Table S1.** Wildlife host, tick species, number and location of adult female *Ixodidae* ticks screened for *Chlamydiales*

QLD: Queensland, NSW: New South Wales, TAS: Tasmania
